# Burn recidivism: a 10-year retrospective study characterizing patients with repeated burn injuries at a large tertiary referral burn center in the United States

**DOI:** 10.1186/s41038-019-0145-4

**Published:** 2019-03-19

**Authors:** Sarah L. Laughon, Bradley N. Gaynes, Lori P. Chrisco, Samuel W. Jones, Felicia N. Williams, Bruce A. Cairns, Gary J. Gala

**Affiliations:** 1 Department of Psychiatry, 101 Manning Drive, CB #7160, Chapel Hill, NC 27599-7160 USA; 2 Department of Surgery, 4001 Burnett-Womack Building, CB #7050, Chapel Hill, NC 27599-7050 USA; 3 North Carolina Jaycee Burn Center, 101 Manning Drive, CB #7206, Chapel Hill, NC 27599-7600 USA

**Keywords:** Burn recidivism, Consult psychiatry, Substance use disorder, Repeat burn injury

## Abstract

**Background:**

Psychiatric and substance use disorders are common among trauma and burn patients and are known risk factors for repeat episodes of trauma, known as trauma recidivism. The epidemiology of burn recidivism, specifically, has not been described. This study aimed to characterize cases of burn recidivism at a large US tertiary care burn center and compare burn recidivists (RCs) with non-recidivists (NRCs).

**Methods:**

A 10-year retrospective descriptive cohort study of adult burn patients admitted to the North Carolina Jaycee Burn Center was conducted using data from an electronic burn registry and the medical record. Continuous variables were reported using medians and interquartile ranges (IQR). Chi-square and Wilcoxon-Mann-Whitney tests were used to compare demographic, burn, and hospitalization characteristics between NRCs and RCs.

**Results:**

A total of 7134 burn patients were admitted, among which 51 (0.7%) were RCs and accounted for 129 (1.8%) admissions. Of the 51 RCs, 37 had two burn injuries each, totaling 74 admissions as a group, while the remaining 14 RCs had between three and eight burn injuries each, totaling 55 admissions as a group. Compared to NRCs, RCs were younger (median age 36 years vs. 42 years, *p* = 0.02) and more likely to be white (75% vs. 60%, *p* = 0.03), uninsured (45% vs. 30%, *p* = 0.02), have chemical burns (16% vs. 5%, *p* <  0.0001), and have burns that were ≤ 10% total body surface area (89% vs. 76%, *p* = 0.001). The mortality rate for RCs vs. NRCs did not differ (0% vs. 1.2%, *p* = 0.41). Psychiatric and substance use disorders were approximately five times greater among RCs compared to NRCs (75% vs. 15%, *p* <  0.001). Median total hospital charges per patient were nearly three times higher for RCs vs. NRCs ($85,736 vs. $32,023, *p* <  0.0001).

**Conclusions:**

Distinct from trauma recidivism, burn recidivism is not associated with more severe injury or increased mortality. Similar to trauma recidivists, but to a greater extent, burn RCs have high rates of comorbid psychiatric and medical conditions that contribute to increased health care utilization and costs. Studies involving larger samples from multiple centers can further clarify whether these findings are generalizable to national burn and trauma populations.

## Background

Trauma, including injuries sustained from motor vehicle collisions (MVCs), falls, burns, gunshot wounds (GSWs), head injuries, stabbings, and more accounts for more years of life lost and disability among Americans than any other disease, including heart disease and cancer [1]. It is among the top three most costly health conditions in the United States, [2] highlighting trauma as a significant public health problem. Among trauma patients, those with psychiatric and/or substance use disorders have been found to require longer hospitalizations, accrue higher hospital costs, have increased rates of complications, and increased mortality when compared to trauma injured patients without psychiatric and/or substance use disorders [3, 4].

Although once considered to be an acute and random event, trauma is widely accepted as a chronic, recurrent, and often preventable condition [5, 6]. Repeat episodes of traumatic injury, known as trauma recidivism, have an average incidence rate of 20% [5, 7–13]. Recurrent injuries requiring medical treatment contribute to growing healthcare costs and increased mortality rates [11, 14, 15]. Consequently, investigators have sought to identify sociodemographic, clinical, and injury-specific patterns among trauma recidivists [12, 16–19] as a means to guide intervention and prevention strategies among this high-risk population. The risk factors most strongly associated with a primary traumatic injury—young age, male gender, lack of insurance, psychiatric disorders, and substance use disorders—are also associated with a primary burn injury [3, 4, 20, 21]. When a patient has comorbid psychiatric and/or substance use disorder, the rate of trauma recidivism has been found to increase two to threefold (40–60%) [5, 6, 22, 23]. Mechanisms of injuries found to be associated with recidivism include GSWs/stabbings [8, 16], falls [7, 23], assaults [16, 23, 24], and MVCs [8, 23]. Previous studies of trauma recidivism, however, generally exclude burn-injured patients [5, 8, 9, 12, 24]; when included, burn-injured patients account for less than 3% of the study population [7, 17, 23, 25]. This low incidence of burn-injured patients in trauma recidivism studies makes it difficult to characterize the patient with repeated burn injury. Given the heterogeneous nature of traumatic injury, the high rate of recurrence, shared risk factors between trauma and burn patients, and an opportunity for intervention, a thorough investigation into recurrent burn injuries is warranted.

We observed patients with repeat burn injuries at our institution. To the best of our knowledge, the phenomenon of burn recidivism, specifically, has not been described. Such a description could help clarify the frequency and costs of this type of recidivism, identify important risk factors for the development of burn recidivism, and guide future research in management and prevention strategies among this subset of burn-injured patients. Accordingly, this study aims to characterize cases of burn recidivism at the North Carolina Jaycee Burn Center, a large tertiary care referral burn center in the southern region of the US, and compare burn recidivists (RCs) with non-recidivists (NRCs).

## Methods

### Setting

This study was conducted at the North Carolina Jaycee Burn Center at the University of North Carolina at Chapel Hill; a large tertiary referral burn center with over 1700 admissions in 2017. It is one of 66 verified burn centers in the US for adult and pediatric patients with burn injuries and adheres to the rigorous guidelines established by the American Burn Association (ABA) and the American College of Surgeons (ACS) [26].

### Study design and patient population

The University of North Carolina Institutional Review Board (IRB) approved this retrospective descriptive cohort study of all adult patients admitted to the North Carolina Jaycee Burn Center for treatment of a burn injury between January 1, 2005, and December 31, 2015 (#15-3094). Patients were identified using the University of North Carolina burn registry, a large electronic database that contains demographic and clinical information on all patients admitted to the North Carolina Jaycee Burn Center from 1994 to present. Patients were excluded from the study if they were admitted for treatment of a cutaneous condition other than a burn, admitted for treatment complications related to a previous burn injury, less than 18 years old at the time of (all) admission(s), or admitted or discharged outside of the identified study period.

### Definitions

**Fig. 1:**
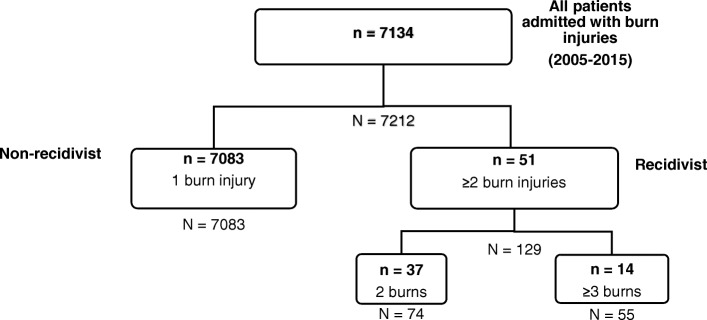
Stratification of all admitted burn patients (*n*) based on the number of burn injury admissions (*N*)

All identified patients admitted for a new burn injury were divided into two groups: RCs, defined as any patient admitted with two or more burn injuries, and NRCs, defined as any patient admitted for one burn injury during the identified study period (Fig. 1). The RC patients (*n*) were further divided into sub-groups based on the total number of new burn injuries (*N*). The RC2s sub-group included patients admitted for only two new burn injuries, RC3s for three burn injuries, RC4s for four, RC5s for five, RC7s for seven, and RC8s for eight burn injuries. The RC3 through RC8s patients were combined into a single group of RC ≥ 3s, defined as patients admitted with three or more burn injuries during the study period. The index burn was defined as the first burn injury a patient had during the identified study period. Thus, any burn injury that occurred before or after the study period was not included. Data was organized according to the number of burn injury admissions to ensure patients with multiple burn injuries were captured.

### Variables of interest

Sociodemographic data included age, gender, race, ethnicity, and primary payer status. Clinical data included the percent total body surface area (TBSA) burned, etiology and characteristics of index and repeat burn(s), time between index and repeat burn(s), presence of inhalation injury, the revised Baux score [27], utilization of and days on mechanical ventilation (MV), utilization of and days in the intensive care unit (ICU), hospital length of stay (LOS), hospital charges, and mortality.

Additional key data, including blood alcohol level (BAL) and urine drug screen (UDS) at admission, was obtained from the electronic medical record (EMR). Any nonzero BAL, measured in milligrams per deciliter (mg/dL) was considered positive. A UDS with amphetamines, benzodiazepines, cannabinoid, cocaine, methadone, or opiates present was considered positive. We were not able to differentiate between positive UDS results that may have been iatrogenic, secondary to the administration of opiates for pain control and benzodiazepines for sedation. Nevertheless, we included all positive UDS results for analysis. The following were considered present if documented in the EMR or burn registry at any time during the study period: comorbid medical conditions, psychiatric disorders (divided into non-substance use and substance use disorders), and tobacco use disorder. A psychiatric consultation was considered to have occurred if it was documented during any admission for a burn injury.

All burn injury characteristics were categorized according to the ABA definitions [28]. Burn etiology was recorded for all admissions and categorized as fire/flame, scald, contact, chemical, electrical, and other/unknown. Burn circumstance was categorized as accident (non-work related or work-related), suspected assault/abuse, suspected self-inflicted, or other/unknown. Hospital costs included all hospital charges billed to the patient and/or the insurance company and did not include physician costs, which are billed separately and not captured in the burn registry.

### Statistical analysis

Data was collected from the burn registry by burn registrar staff, including one of the authors (LC) and from the electronic medical record by one of the authors (SL) and then compiled into a secure spreadsheet. Continuous variables were reported using the median and interquartile ranges (IQR) with the 25th and 75th percentiles. Categorical variables and continuous variables were analyzed using chi-square and Wilcoxon-Mann-Whitney test, respectively, with significance levels set at 5% (*p* <  0.05). All analyses were performed using SAS 9.4 (SAS Inc., Cary, NC).

## Results

Between January 1, 2005, and December 31, 2015, a total of 7134 adult patients were hospitalized for a burn injury; 51 (0.7%) patients were identified as RCs, accounting for 1.8% (129/7212) of the total admissions and the remaining 7083 patients as NRCs (Fig. 1). Among all 129 admissions for burn injuries by RCs, 43% (55/129) of admissions were attributable to RC ≥ 3s and 57% (74/129) to RC2s. The median time between burn incidents was 10 months (IQR, 2.9–36.3). The hospital charges of all 51 RCs with 129 burn injury admissions totaled nearly 8 million dollars. There were no burn mortalities among RCs and few burn mortalities (0 vs 1.2%, *p* = 0.41) among NRCs.

### Patient demographics

Compared to NRCs, RCs were younger [median age 36 years (IQR 25–48) vs. 42 years (IQR 29–55), *p* = 0.02] and more likely to be white (75% vs 60%, *p* = 0.03), non-Hispanic (100% vs 91.5%, *p* = 0.03), and uninsured (45% vs 30%, *p* = 0.02). Standard sociodemographic measures of the RCs compared with the larger NRC group are detailed in Table 1.

**Table 1:** Patient demographics based on the number of burn injury admissions to the North Carolina Jaycee Burn Center, 2005–2015

	Non-recidivist (NRC) *n* = 7083	Recidivist (RC) *n* = 51	RC2s *n* = 37	RC≥3 *n* = 14	*p* value NRC vs. RC
Age, years, median (IQR)	42 (29–55)	36 (25–48)	37 (27–44)	39.5 (36–52)	0.02*
Male	5116 (72.2)	35 (68.6)	25 (67.6)	10 (71.4)	0.57
Female	1967 (27.8)	16 (31.4)	12 (32.4)	4 (28.6)	0.57
Race, *n* (%)					
Black	1923 (28.5)	11 (21.5)	10 (27.0)	1 (7.1)	0.27
White	4030 (59.7)	38 (74.5)	26 (70.3)	12 (85.7)	0.03*
Other^a^	792 (11.7)	2 (3.9)	1 (2.7)	1 (7.1)	0.08
Unknown	338	0	0	0	
Ethnicity, *n* (%)					
Non-hispanic	5349 (91.5)	51 (100)	37 (100)	14 (100)	0.03*
Hispanic	496 (8.5)	0	0	0	0.03*
Unknown	1238	0	0	0	
Primary payer, *n* (%)					
Private	2077 (29.6)	9 (17.6)	7 (18.9)	2 (14.3)	0.06
Self-pay	2118 (30.2)	23 (45.1)	17 (45.9)	6 (42.9)	0.02*
Medicaid	915 (13.0)	10 (19.6)	5 (13.5)	5 (35.7)	0.16
Medicare	1141 (16.3)	7 (13.7)	7 (18.9)	0	0.62
Other^b^	761 (10.9)	2 (3.9)	1 (2.7)	1 (7.1)	0.17
Unknown	71	0	0	0	

*IQR* interquartile range (25th–75th)

NRC, patients with only 1 burn injury admission

RC, all recidivists (RC2s + RC≥3)

RC2s, patients with exactly 2 burn injury admissions

RC≥ 3, patients with 3 or more burn injury admissions (range 3–8)

^a^Other includes American Indian/Alaska Native, Asian, Native Hawaiian or Other Pacific Islander

^b^Other includes workers’ compensation, military, or other government-issued insurance

*p < 0.05

### Burn injury characteristics

A pattern of smaller, less severe burns, with lower rates of inhalation injury, was noted among RCs compared to NRCs (Table 2). Of all burn injuries among RCs, nearly 90% (115/129) had burns ≤ 10% TBSA [median 2% (IQR, 1–4)] and 57% (73/129) were first or second-degree burn injuries. Chemical burns accounted for approximately three times more burns among RCs than NRCs and of the RCs with a chemical burn, 76% (16/21) were RC ≥ 3. In almost half [45% (23/51)] of RCs, the burn etiology and anatomical location of the burn injury were the same for at least two burn injuries, if not more. Burn injuries exclusively located on patients’ extremities were common among RCs, with extremity burns alone accounting for 83% (107/129) of all RC burn injury admissions. Self-inflicted burn injuries were suspected in less than 1% (59/7083) of NRCs versus 21.7% (28/129) of RCs, *p* <  0.0001. During at least one, but sometimes numerous admission(s) for a burn injury among RC ≥ 3, there was suspicion for self-inflicted burn injury in 86% (12/14) of the group.

**Table 2:** Burn characteristics and hospital service use of burn patients (*n*) based on the number of burn injury admissions (*N*) to the North Carolina Jaycee Burn Center, 2005–2015

	Non-recidivist (NRC) *n* = 7083	Recidivist (RC) *n* = 51	RC2s *n* = 37	RC≥ 3 *n* = 14	*p* value NRC vs. RC
Burn injury admissions, *N*	7083	129	74	55	n/a
Burn etiology, *N* (%)					
Flame	3583 (52.6)	45 (34.9)	33 (44.6)	12 (21.8)	0.0001*
Scald	2178 (32.0)	42 (32.6)	27 (36.4)	15 (27.3)	0.89
Contact	402 (5.9)	19 (14.7)	7 (9.5)	12 (21.8)	< 0.0001*
Chemical	373 (5.5)	21 (16.3)	5 (6.8)	16 (29.0)	< 0.0001*
Electrical	255 (3.7)	2 (1.6)	2 (2.7)	0	0.22
Radiation	19 (0.3)	0	0	0	0.53
Unknown	273	0	0	0	
Burn circumstance, *N* (%)					
Accident, non-work	5862 (83.6)	93 (72.0)	63 (85.1)	30 (54.5)	0.001*
Accident, work-related	997 (14.2)	2 (1.6)	2 (2.7)	0	< 0.0001*
Suspected self-inflicted	59 (0.8)	28 (21.7)	7 (9.5)	21 (38.2)	< 0.0001*
Suspected assault/abuse	94 (1.3)	6 (4.7)	2 (2.7)	4 (7.3)	0.001*
Unknown	71	0	0	0	
Burn severity, *N*					
TBSA, median (IQR)	3.5 (1–8)	2.0 (1–4)	3.0 (1–6)	1.0 (1–3)	< 0.0001*
≤ 10% TBSA, *N* (%)	5384 (76.0)	115 (89.1)	63 (85.1)	52 (94.5)	0.001*
Revised Baux, median (IQR)^a^	48.8 (35–63)	42.5 (33–55)	42.3 (29–57)	44.2 (38–52)	0.001*
Inhalation injury present, *N* (%)	479 (6.8)	7 (5.4)	7 (9.5)	0	0.53
Outcome variables					
LOS, days, median (IQR)	7 (2–14)	7 (2–13)	8 (4–15)	6 (2–11)	0.88
Required ICU, *N* (%)	2311 (32.6)	27 (20.9)	21 (28.4)	6 (10.9)	0.005*
ICU, days, median (IQR)	3 (1–14)	5 (1–16)	5 (2–22)	4 (1–7)	0.81
Required MV, *N* (%)	931 (13.1)	10 (7.8)	10 (13.5)	0	0.08
MV, days, median (IQR)	9 (2–38)	8 (2–33)	8 (2–33)	n/a	0.94
Mortality, *n* (%)	82 (1.2)	0	0	0	0.41

*IQR* interquartile range (25th–75th), *TBSA* total burn surface area, *LOS* length of stay, *ICU* intensive care unit, *MV* mechanical ventilation

NRC, patients with only 1 burn injury admission

RC, all recidivists (RC2s + RC≥ 3)

RC2s, patients with exactly 2 burn injury admissions

RC≥ 3, patients with 3 or more burn injury admissions (range 3–8)

^a^Revised Baux score = age + % TBSA + 17 × (inhalation injury, 1 = yes, 0 = no)

*p < 0.05

### Hospital service use characteristics

RCs compared to NRCs had similar lengths of hospitalizations [median 7 (IQR, 2–13) days; range 1–140 days vs 7 (IQR, 2–14) days; range 1–412 days], but lower rates of ICU (21% vs 33%, *p* = 0.005) and MV (8% vs 13%, *p* = 0.08) utilization (Table 2). Among RCs and NRCs requiring ICU level care and/or MV, the time utilizing each was similar across both groups. The total hospital charges for all 51 burn RCs with 129 admissions was $7,813,075. The median hospital charges *per admission* was similar for RCs ($30,882 per 129 admissions) and NRCs ($32,023 per 7083 admissions, *p* = 0.78). However, further analysis of hospital charges on a *per patient* basis revealed the median total hospital charge for each of the 51 RCs was $85,736 (IQR, $45,089–$149,602), nearly three times as much than the median total hospital charge for each of the 7083 NRCs of $30,882 (IQR, $87,123–$52,228), *p* = 0.0001.

### Other key clinical data

Slightly more than half of all patients received a UDS (Table 3). Opioids were the most commonly detected substance in both groups though the overall percentage was higher among RCs compared to NRCs (82% vs 65.2%, *p* = 0.003). The following specific substances were detected in the UDS of RCs vs NRCs, respectively: cannabis (36% vs 26%, *p* = 0.056), benzodiazepines (37.5% vs 19.4%, *p* = 0.0001), and cocaine (15.3% vs 10.5%, *p* = 0.19). Furthermore, among all patients with a positive UDS, two or more substances were detected in 65.7% (44/67) of RCs compared with 44.9% (1341/2984) of NRCs, *p* = 0.0007. For those with a positive BAL, the mean BALs did not differ between RCs [145 mg/dL (± 134)] and NRCs [136 mg/dL (± 118)], *p* = 0.82.

**Table 3:** Clinical key data of burn patients (*n*) based on the number of burn injury admissions (*N*) to the North Carolina Jaycee Burn Center, 2005–2015

	Non-recidivist (NRC) *n* = 7083 *N* = 7083	Recidivist (RC) *n* = 51 *N* = 129	RC2 *n* = 37 *N* = 74	RC≥ 3 *n* = 14 *N* = 55	*p* value NRC vs. RC
Psychiatric consult, *n* (%)	628 (8.9)	11 (21.6)	11 (29.7)	6 (42.9)	< 0.0001*
Alcohol and drug screening, *N* (%)					
UDS obtained	3665 (51.7)	72 (55.8)	47 (63.5)	25 (45.5)	0.36
UDS positive^a^	2984 (81.4)	67 (93.1)	42 (89.4)	25 (100)	0.01*
BAL obtained	3445 (48.6)	59 (45.7)	40 (54.1)	19 (34.5)	0.51
BAL positive^b^	408 (11.8)	9 (15.3)	5 (12.5)	4 (21.1)	0.41
Comorbidities, *n* (%)					
Psychiatric disorder	1034 (14.6)	38 (74.5)	24 (64.9)	14 (100)	< 0.0001*
Substance use disorder	881 (12.4)	39 (76.5)	26 (70.3)	13 (92.9)	< 0.0001*
Tobacco use disorder	1018 (14.4)	43 (84.3)	29 (78.4)	14 (100)	< 0.0001*

*UDS* urine drug screen, *BAL* blood alcohol level

NRC, patients with only 1 burn injury admission

RC, all recidivists (RC2s + RC***≥*** 3)

RC2s, patients with exactly 2 burn injury admissions

RC***≥*** 3, patients with 3 or more burn injury admissions (range 3–8)

^a^Positive includes amphetamines, cocaine, cannabis, opiates, benzodiazepines, and barbiturates

^b^Positive includes any non-zero BAL

*p < 0.05

### Comorbidities

**Fig. 2:**
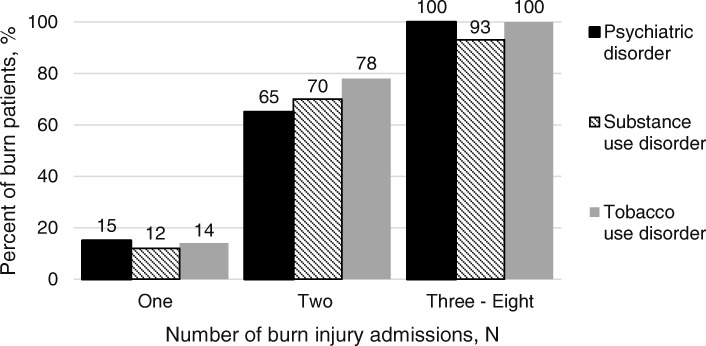
Psychiatric comorbidities among burn patients (*n*) based on the number of burn injury admissions (*N*)

A comorbid psychiatric disorder was present in 75% (38/51) of RCs, five times more than in the NRC group, *p* <  0.0001 (Fig. 2). Among RCs with mental illness, 78.9% (30/38) had two or more psychiatric disorders. Trauma- and stressor-related disorders [posttraumatic stress disorder (PTSD) or acute stress disorder (ASD)] and depressive disorders (major depressive disorder or unspecified depressive disorder) were the most common categories of psychiatric diagnoses among RCs, present in 47.4% (18/38) and 68.4% (26/38) of all RCs, respectively. Among NRCs with a psychiatric disorder, 28.9% (299/1034) had a depressive disorder and 21.6% (223/1034) had an anxiety disorder. PTSD and ASD accounted for just 6.7% (69/1034) of NRCs with a mental illness.

Opioids were the most commonly abused substance among all RCs. Opioid use disorder accounted for more than 50% (29/51) of the substance use disorders in the RC group but was present in less than 1% (50/7083) of NRCs, *p* <  0.0001. Though substance use disorders were less common among NRCs compared to RCs, nearly 50% (426/881) of NRCs with a substance use disorder had an alcohol use disorder. The proportion of RC ≥ 3 and RC2s with cocaine (29% vs 32%, *p* = 0.84), cannabis (36% vs 46%, *p* = 0.52), and alcohol use disorder (42.9% vs 43.2%, *p* = 0.98) was similar. In contrast, more RC ≥ 3 than RC2s had opioid (79% vs 49%, *p* = 0.056) and benzodiazepine (43% vs 27%, *p* = 0.28) use disorder, respectively. Of the 16 RCs with benzodiazepine use disorder, all (16/16) had concurrent opioid use disorder. Furthermore, among the 92.9% (13/14) of RC ≥ 3 with a substance use disorder, all (13/13) had two or more substance use disorders.

All RC ≥ 3 (14/14) and the majority of RC2s (27/37) had at least two comorbid medical conditions with cardiovascular disease (hypertension, hyperlipidemia, coronary artery disease, congestive heart failure), seizure disorder, diabetes mellitus, and chronic obstructive pulmonary disease being the most common. Chronic pain, considered distinct from other medical conditions in our study, was comorbid in 65% (33/51) of all RCs [79% (11/14) of RC ≥ 3 vs 59% (22/37) of RC2s]. Unfortunately, comparison data for these parameters was unavailable for the larger NRC group.

## Discussion

Nearly three decades of literature on trauma recidivism exists. To date, however, information on recidivism among patients injured by burns is limited. To our knowledge, burn recidivism specifically has not been previously identified or described in the trauma and burn literature. Our study introduces formative data about burn recidivism and its epidemiology. During the 10-year study period, the incidence of burn recidivism at our institution was approximately 1% and accounted for nearly 2% of all adult burn-injury admissions. Previous rates of trauma recidivism from any cause range from 0.4 to 45% [5–15, 18, 22–24, 29–31]; this variability in rates of recidivism reflects a significant heterogeneity among these studies’ design, methodology, time frame, and population. Our rate of 1% is on the lower end of the range of recidivism described in the general trauma recidivism literature. This relatively low percentage rate may be explained by limiting the study sample to only admitted patients at a single burn center. RCs with burn injuries who presented to other institutions and/or other types of health care settings (e.g., emergency departments, outpatient clinics, and urgent care centers) would not have been captured under our study design. Thus, overall, this rate likely is an underrepresentation of the true incidence of burn recidivism.

Trauma recidivism has generally been associated with increased mortality, morbidity, healthcare utilization, and costs [5, 12, 13, 22, 29, 32–34]. Investigations of all-cause trauma recidivists have revealed mortality rates upwards of 7.5% [35]. Among penetrating trauma recidivists, one study found the likelihood of mortality increased twofold with each new penetrating trauma incident and the 5-year mortality rate was 20% [12]. In contrast to trauma recidivism, the mortality among all burn RCs in our study was zero. Furthermore, RCs had smaller burns and lower rates of inhalation injury compared to NRCs. In fact, there were no inhalation injuries or use of MV during any of the 55 burn injury admissions by RC ≥ 3. Not surprisingly, RCs were less likely to utilize the ICU and require MV, suggesting lower morbidity compared to NRCs. While we found burn RCs differed from trauma recidivists with regards to lower rates of mortality and morbidity, the cumulative effect from repeated hospitalizations among burn RCs at our institution resulted in increased healthcare utilization and costs, similar to data published on trauma recidivists. Individually, each of the 51 RCs had total median hospital charges of $85,736. These hospital charges are approximately three times those of the individual NRC median charge of $32,023, *p* <  0.0001. Our findings suggest the phenomenon of burn recidivism is contributing to increased healthcare expenditures.

Burn RCs, similar to trauma recidivists were more likely to be male, young, white, non-Hispanic, and uninsured (Table 1) when compared to NRCs. In addition, we found burn RCs had high rates of psychiatric and substance use disorders, known risk factors associated with trauma recidivism. The prevalence of psychiatric disorders among trauma recidivists is estimated to range from 20 to 42% [8, 10, 11, 16, 20, 23, 36] and substance use disorders to be as high as 67% [5, 7–10]. In line with trauma recidivism, but to an even greater extent, our study revealed high prevalence rates of comorbid psychiatric and substance use disorders among burn RCs (Fig. 2). Among RC ≥ 3, all (14/14) had a mental illness, 86% (12/14) had two or more psychiatric diagnoses, and 93% (13/14) had both psychiatric and substance use disorders. These findings highlight the importance of a multidisciplinary approach to burn-injured patients, including the early involvement of a psychiatric consultant particularly when the patient has had a previous burn injury.

Self-inflicted injuries have been associated with trauma recidivism [34, 37, 38] and burn injuries [39–43]; generally, this population has significant psychiatric histories, with a high prevalence of personality, substance use, and psychotic disorders. In addition, high-risk behaviors and substance abuse, both associated with low-risk perception and high impulsivity, have been found to contribute to repeat injuries [44, 45]. While some repeated burn injuries among RCs, namely RC2s, occurred in the context of continued high-risk behaviors and/or untreated mental illness, a subset, particularly among RC ≥ 3 appeared to be strongly linked with aberrant drug-seeking behaviors. Among all burn recidivists, only 2% (1/51) endorsed self-inflicting a burn injury with the intention of causing death and/or disability. In contrast, 86% (12/14) of RC ≥ 3 were suspected of self-inflicting burn injuries despite reporting the circumstance surrounding the burn-injury as accidental; among these, 58% (7/12) were strongly suspected of being motivated by a desire for opioids and benzodiazepines. Indeed, since completion of this project’s data collection in December 2015, we have observed 20 additional burn recidivists at our institution; nearly half of these RCs having at least one burn injury suspicious for being self-inflicted and motivated by a desire to receive opioids ± benzodiazepines. This type of deceptive intentionality strongly linked with the desire to obtain opioids and benzodiazepines appears unique among burn recidivists. Further research is needed to better understand this phenomenon of deceptive self-burning for opioids and benzodiazepines, particularly in light of the opioid epidemic.

There are limitations inherent of retrospective studies that are applicable to this study. Some of the elements obtained from the burn database were incomplete. We attempted to mitigate this limitation by individually reviewing each patients’ EMR to verify and correct, when indicated, any element obtained by the registry; however, the size of the NRC group made this challenging. For instance, medical comorbidities, including chronic pain were not included in the burn registry until 2008 and were therefore unavailable for the large NRC group. As the presence of comorbidities have been found to be associated with increased burn mortality [46–48], specific details about the type and number of comorbid medical condition(s) among burn NRCs compared to burn RCs could help further characterize this population and also determine if there is variability among different types of recidivism. Additionally, information regarding marital, environmental, homeless, and employment status is limited in our registry; these variables have been associated with trauma recidivism in some studies [8, 10, 13, 16, 23, 49] and, if available, could expand our knowledge on burn recidivism. A psychiatric consultation was requested and provided among nearly 60% of RCs compared with less than 10% of NRCs. It is reasonable to wonder whether more psychiatric consults in the NRC group may have resulted in a higher prevalence of psychiatric and substance use disorders among the NRC group. Our study was limited to data from a single large, tertiary burn center that focused on burn injuries associated with hospitalization. This has implications regarding the generalizability of our findings and most likely highlights that the recidivism rate of nearly 1% is an underrepresentation of the true incidence of burn recidivism that would be obtained if a nationwide cohort were examined.

## Conclusions

We hope that this study will raise awareness into the phenomenon of burn recidivism and serve as a platform for future research within the area of burn recidivism. Utilizing a national large-scale database that captures a diverse patient population, various geographic regions, and different types of medical encounters (e.g., visits to emergency departments, urgent care centers and physician offices), in addition to hospital admissions to both burn and non-burn centers, would expand our knowledge of burn recidivism and its true burden on society. Identifying patterns and characteristics of burn recidivists will allow providers to anticipate and intervene on those particular problems that contribute to burn recidivism, as well as implement appropriate treatment, and prevention measures. There is a growing body of evidence to support brief psychiatric and substance use interventions as effective in reducing the rate of recidivism and associated costs among trauma patients [50–54]. Similarly, interventions developed and aimed at targeting specific high-risk trauma recidivist populations have been implemented with some successful results [51, 55, 56]. A collaborative and multidisciplinary approach involving burn surgeons, general and trauma surgeons, emergency department physicians, consult-liaison psychiatrists, general psychiatrists, and addiction specialists could help to develop both prevention and intervention strategies to better address the issue of burn recidivism.

## Abbreviations

ABA: American Burn Association
ACS: American College of Surgeons
BAL: Blood alcohol level
EMR: Electronic medical record
ICU: Intensive care unit
LOS: Length of stay
MV: Mechanical ventilation
NRC: Non-recidivist
RC≥ 3 s: Patients with 3 or more burn injury admissions
RC: Recidivist
RC2s: Patients with exactly 2 burn injury admissions
TBSA: Total body surface area
UDS: Urine drug screen
